# Inflammatory breast cancer shows angiogenesis with high endothelial proliferation rate and strong E-cadherin expression

**DOI:** 10.1038/sj.bjc.6600807

**Published:** 2003-03-04

**Authors:** C G Colpaert, P B Vermeulen, I Benoy, A Soubry, F Van Roy, P van Beest, G Goovaerts, L Y Dirix, P Van Dam, S B Fox, A L Harris, E A Van Marck

**Affiliations:** 1 Department of Pathology, University Hospital Antwerp, University of Antwerp, B-2650 Edegem, Belgium; 2 Angiogenesis Group, AZ Sint Augustinus, B-2610 Antwerp, Belgium; 3 Department for Molecular Biomedical Research, Flanders Interuniversity Institute for Biotechnology (VIB), Ghent University, B-9000 Ghent, Belgium; 4 Weatherall Institute of Molecular Medicine, John Radcliffe Hospital, Headington, Oxford OX39DS, UK

**Keywords:** angiogenesis, E-cadherin, fibrin, hypoxia, inflammatory breast cancer

## Abstract

Inflammatory breast cancer (IBC) is the most aggressive form of breast cancer. Improved understanding of the mechanisms responsible for the differences between IBC and non-IBC might provide novel therapeutic targets. We studied 35 consecutive patients with IBC, biopsied prior to the initiation of chemotherapy. Angiogenesis was evaluated by Chalkley counting and by assessment of endothelial cell proliferation (ECP) and vessel maturity. The presence of fibrin, expression of the hypoxia marker carbonic anhydrase IX (CA IX) and epithelialcadherin (E-cadherin) expression were immunohistochemically detected. The same parameters were obtained in a group of 104 non-IBC patients. Vascular density, assessed by Chalkley counting (*P*<0.0001), and ECP (*P*=0.01) were significantly higher in IBC than in non-IBC. Abundant stromal fibrin deposition was observed in 26% of IBC and in only 8% of non-IBC (*P*=0.02). Expression of CA IX was significantly less frequent in IBC than in non-IBC with early metastasis (*P*=0.047). There was a significant positive correlation between the expression of CA IX and ECP in IBC (*r*=0.4, *P*=0.03), implying that the angiogenesis is partly hypoxia driven. However, the higher ECP in IBC and the less frequent expression of CA IX in IBC *vs* non-IBC points at a role for other factors than hypoxia in stimulating angiogenesis. Strong, homogeneous E-cadherin expression was found at cell–cell contacts in all but two IBC cases, both in lymphovascular tumour emboli and in infiltrating tumour cells, challenging our current understanding of the metastatic process. Both the intense angiogenesis and the strong E-cadherin expression may contribute to the highly metastatic phenotype of IBC.

Inflammatory breast cancer (IBC) constitutes less than 10% of all breast cancers (Chevallier *et al*, 1987; Fields *et al*, 1989; Alpaugh *et al*, 1999). It is a distinct and most aggressive form of locally advanced malignant breast disease. Although multimodality treatments have achieved significant improvements in both disease-free and overall survival (Chambler *et al*, 1995), prognosis is still extremely poor because of the high incidence of local recurrence and systemic metastases. Very little is known about the pathogenesis of IBC. Only few studies have examined the differences between IBC and non-IBC (Aziz *et al*, 2001). However, a better understanding of the effector molecules responsible for the IBC phenotype might provide important new therapeutic targets.

Recently, using a semiquantitative reverse transcriptase polymerase chain reaction, Shirakawa *et al* (2002b) showed that angiogenic, but not lymphangiogenic factors are overexpressed in resected specimens of 19 IBC patients when compared with 23 non-IBC tumours. In the same study, a significantly higher population of tumour-infiltrating endothelial cells or endothelial precursor cells was immunohistochemically found in the tumour-associated stroma of IBC specimens than in non-IBC specimens. The authors conclude that IBC tumours induce angiogenesis and vasculogenesis that involves recruitment of endothelial cells or endothelial precursor cells. They further studied the pathways involved in IBC angiogenesis in an IBC xenograft model (WIBC-9) originating from a patient with IBC. Histologically, WIBC-9 xenografts exhibited invasive ductal carcinoma with angiogenesis at the tumour margin and vasculogenic mimicry with absence of endothelial cells in addition to necrosis and fibrosis in the tumour centre (Shirakawa *et al*, 2002a). To the best of our knowledge, however, quantitative morphologic studies of angiogenesis in human IBC have not yet been performed.

Another well-studied xenograft model of IBC is the human-severe combined immunodeficient/nude mice model MARY-X (Alpaugh *et al*, 1999; Tomlinson *et al*, 2001, Alpaugh *et al*, 2002). Comparative studies of MARY-X with non-IBC xenografts indicated a 10–20-fold overexpression of E-cadherin. Cadherins are a family of transmembranous glycoproteins responsible for calcium-dependent, homophilic intercellular adhesion (Takeichi, 1991). Epithelial cadherin (E-cadherin) is present in most normal adult epithelial tissues and plays a critical role in the maintenance of normal tissue architecture. Tomlinson *et al* (2001) studied 25 cases of human IBC and found E-cadherin membrane immuno-reactivity in all cases. Kleer *et al* (2001) found strong expression of E-cadherin in 100% of 20 human IBC tumours and in only 68% of 22 stage-matched non-IBC tumours. IBC therefore constitutes an important exception to the association between loss of E-cadherin expression and increased metastatic potential and poor outcome in breast cancer. Indeed, the E-cadherin-mediated cell adhesion system is known to act as an invasion suppressor system in cancer cells (Vleminckx *et al*, 1991) and diminished E-cadherin expression has been related to invasiveness, dedifferentiation, lymphovascular invasion, lymph node metastasis, distant metastasis and shortened disease-free survival (Oka *et al*, 1993; Jones *et al*, 1996; Siitonen *et al*, 1996; Asgeirsson *et al*, 2000; Heimann *et al*, 2000). The strong E-cadherin expression in IBC, being a highly invasive, poorly differentiated tumour with prominent lymphovascular invasion and high incidence of lymph node and distant metastasis, therefore constitutes a striking difference between IBC and non-IBC.

The aim of this study was to provide quantitative morphologic data on angiogenesis in human IBC and to confirm the aberrant E-cadherin expression in a group of 35 IBC patients.

## PATIENTS AND METHODS

### Patients

We studied 35 consecutive patients with newly diagnosed primary IBC of whom sufficient formalin-fixed, paraffin-embedded tissue was available, biopsied prior to the initiation of chemotherapy. Mean patient age was 55.6 years (range: 25–83 years). All patients showed diffuse enlargement of the involved breast of sudden onset (all less than 8 weeks, median 5 weeks). There was erythema and oedema of the skin involving more than one-third of the breast. In some patients, a poorly circumscribed tumour was palpable. There was no tumour fungation or skin ulceration. The material consisted of 32 large incisional breast biopsies with overlying skin and of three trucut biopsies. Dermal lymphovascular permeation was found at the time of initial diagnosis in 23 patients (66%), which is consistent with literature data (Bonnier *et al*, 1995). In three additional patients, dermal lymphatic tumour emboli were found at the time of mastectomy, yielding a pathological confirmation of the clinical diagnosis in 74% of the cases. Parenchymal lymphatic invasion was commonly seen.

The haematoxylin–eosin stained slides were reviewed to select the paraffin block best suitable for additional immunohistochemical stains. All selected blocks contained both infiltrative carcinoma and lymphovascular tumour emboli, dermal or parenchymal, except for one trucut biopsy in which lymphovascular permeation was not present.

### Microvessel density (Chalkley count)

Rehydrated paraffin sections were immunostained with a monoclonal antibody against CD34 (clone QBEnd/10, Biogenex, San Ramon, USA) diluted 1 : 5 with overnight incubation at 4°C and without any antigen retrieval procedure. A biotinylated secondary antibody, avidin–peroxidase as an enzyme and diaminobenzidine tetrahydrochloride (DAB) as a chromogenic substrate were used to visualise binding of the first antibody. Microvessel density was determined with the Chalkley method (Chalkley, 1943). This is a morphometric point counting system using a microscope eyepiece graticule and has been suggested as a standard in international consensus reports on quantification of angiogenesis (Vermeulen *et al*, 1996,^2002^). The areas of highest vascular density (‘hot spots’) were identified at low magnification (× 10 ocular and × 10 objective). On a higher magnification (× 10 ocular and × 20 objective, Chalkley grid area 0.22 mm^2^), a 25-point Chalkley eyepiece graticule was applied to each hot spot and oriented to permit the maximum number of points to hit on or in an immunostained microvessel. Chalkley counting was performed in a mean number of seven (range 3–13) hot spots per slide. The Chalkley count was expressed as ‘Chalkley max’, the count in the hot spot with highest vascular density and ‘Chalkley mean’, the mean of the three highest counts.

### Endothelial cell proliferation

For the quantification of proliferating endothelial cells, a proliferating cell nuclear antigen (PCNA)-CD34 double-labelling immunohistochemical staining was performed. A monoclonal antibody directed against PCNA (PC10, Medac, Wedel, Germany; prediluted) was applied to the rehydrated paraffin sections and allowed to incubate overnight at room temperature. A secondary antibody linked to peroxidase and DAB were used to visualise binding of the first antibody. The sections were then incubated overnight at 4°C with an antibody against CD34 (clone QBEnd/10, Biogenex, San Ramon, CA, USA) diluted 1 : 5. The staining was completed with the Ventana Basic Alkaline Phosphatase Red detection kit (Ventana Medical Systems, Inc., Strasbourg, France) on the Ventana NexES automated immunostainer.

The fraction of proliferating endothelial cells (ECP%) was assessed in the areas of highest vascular density (‘hot spots’), identified at low magnification (× 10 ocular and × 10 objective). On a higher magnification (× 10 ocular and × 40 objective), a total number of 100 endothelial cells were evaluated per hot spot and ECP% was the number of endothelial cells with PCNA-stained nuclei per 100 endothelial cells. ECP% max is the fraction of proliferating endothelial cells in the hot spot with the highest endothelial cell proliferation and ECP% mean is the mean of the three highest fractions.

### Blood vessel maturation and microvessel density (Weidner method)

For the assessment of blood vessel maturation, another double-labelling immunohistochemical technique was used to simultaneously stain endothelial cells (CD34) and mural cells (*α*-smooth muscle actin). Paraffin sections were incubated with an antibody to CD34 (clone QBEnd/10, Biogenex, San Ramon, CA, USA; 1 : 5 dilution; overnight incubation) without prior antigen retrieval. A secondary antibody linked to peroxidase followed by DAB was used to visualise the CD34 antigen. The slides were then placed on the Ventana NexES automated immunostainer and stained with an antibody against *α*-smooth muscle actin (clone 1A4, Biogenex, San Ramon, CA, USA; 1 : 80 dilution, 32 min) and the Ventana Basic Alkaline Phosphatase Red detection kit (Ventana Medical Systems, Inc.). Blood vessel immaturity was again assessed in the vascular hot spots, identified at low magnification (× 10 ocular and × 10 objective). On a higher magnification (× 10 ocular and × 25 objective; microscopic field area 0.42 mm^2^), the percentage of immature vessels was calculated as the number of CD34-labelled blood vessels not associated with *α*-smooth muscle actin positive mural cells (pericytes and smooth muscle cells)/total number of blood vessels in the microscopic field.

This total number of vessels also provided us with a quantification of microvessel density according to the method described by Weidner *et al* (1991).

### Fibrin

The presence of fibrin was detected immunohistochemically with the NYB.T2G1 monoclonal antibody (Accurate Chemical and Scientific Corporation, Westbury, NY, USA), which reacts with the amino-terminal part of the B*β* chain only after removal of fibrinopeptide B by thrombin, and hence binds to fibrin but not fibrinogen. The staining was performed on the Ventana NexES automated immunostainer. After protease digestion for 6 min (Sigma p4789 type XXVVII, Sigma-Aldrich, Bornem, Belgium), the primary antibody was incubated for 32 min at a dilution 1 : 100. Binding of the first antibody was visualised with a secondary antibody linked to peroxidase and with DAB, as described above. The presence of fibrin was scored semiquantitatively (abundant: fibrin easily seen at low magnification (× 40), sparse: small deposits of fibrin only visible at higher magnification (> × 100), absent: no fibrin).

### Carbonic anhydrase IX (CA IX)

Immunohistochemical staining for the endogenous hypoxia marker CA IX was performed at the Weatherall Institute of Molecular Medicine in Oxford with the murine monoclonal antibody M75 at a dilution of 1 : 50. The ability of this antibody to specifically detect CA IX expression in tissue sections was previously confirmed by direct correlation with Western blot analysis in human breast tumour specimens (Wykoff *et al*, 2001). After an incubation time of 60 min at room temperature, a peroxidase conjugated to goat anti-mouse immunoglobulins (Dako Envision+System, peroxidase, mouse; Dako, Glostrup, Denmark) was allowed to incubate for 30 min. Slides were then stained with DAB and counterstained with haematoxylin. The immunostaining was quantified both in the infiltrative tumour and in the lymphovascular tumour emboli, by semiquantitative scoring as previously described (Chia *et al*, 2001). In brief, a score of 0–3 for the intensity of staining was given (0: no staining, 1: weak staining, 2: moderate staining, 3: strong staining). The percentage of immunostained tumour cells was estimated. The product (intensity score × the percentage of immunoreactive tumour cells) yielded a final score of 0–300. For statistical evaluation, any percentage of immunostaining was called positive.

### E-cadherin

Immunohistochemical staining for E-cadherin was performed with the murine monoclonal antibody HECD-1 (Human Epithelial Cadherin-1, Takara, Kyoto, Japan). Rehydrated paraffin sections were pretreated for 30 min in an EDTA-buffer (pH 9; DAKO PP20-0226) at 96°C. The primary antibody was incubated for 60 min at a concentration of 1.3 *μ*g ml^−1^. The staining was completed with a biotinylated secondary antibody, avidin–peroxidase and DAB. E-cadherin expression was assessed semiquantitatively. The percentage of tumour cells showing E-cadherin expression at cell-cell contacts was estimated and the intensity of staining was compared with the staining of normal epithelia such as the epidermis and normal mammary ducts (1: weaker than normal structures, 2: staining intensity similar to normal structures, 3: stronger than normal structures). The product (intensity score × the percentage of immunoreactive tumour cells) yielded a final score ranging from 0 to 300. A separate score was given for the infiltrative tumour and the lymphovascular tumour emboli.

### Noninflammatory breast cancer patients (non-IBC)

For comparison, the same parameters were assessed in a group of 104 non-IBC patients, which we studied before (Colpaert *et al*, 2001a). This group consisted of lymph node-negative breast cancer patients, with an unfavourable subgroup of 46 consecutive patients who developed early distant relapse after a median disease-free survival of 25 months, and a favourable subgroup of 58 patients who were still disease-free after a median follow-up of 91.5 months. The rapid clinical progression of the non-IBC patient group with early distant metastasis makes it a relevant control group for the IBC patients from a clinical point of view.

None of the non-IBC patients received systemic adjuvant therapy. Mean age at diagnosis was 53±10.7 years for the unfavourable subgroup and 55.9±10.4 years for the favourable subgroup. Tumour size was 2.02±0.79 cm in the former and 1.69±0.83 cm in the latter group. A total of 42% of the tumours with unfavourable outcome and only 14% of the tumours with favourable outcome were of high histological grade.

Most parameters were assessed on the total patient group. For endothelial cell proliferation and vessel maturation, 16 patients from the unfavourable group and 18 patients from the favourable group were randomly selected.

### Statistics

Results were analysed for statistical significance by the Mann–Whitney *U*-test or the Kruskal–Wallis test for continuous variables in different subgroups. The χ^2^-square test was used for testing relations between categorical variables and correlation analysis was used for continuous variables. A *P*-value <0.05 was required for significance. Statistical calculations were performed with the Statview 4.51 program (Abacus Concepts; Apple Macintosh).

## RESULTS

### Microscopical phenotype

The microscopical phenotype was evaluated on the haematoxylin–eosin stained slides. All IBC had a diffusely infiltrative growth pattern. There was no well-circumscribed tumour. Instead, the carcinoma infiltrated between pre-existing structures such as mammary lobules and ducts, often with the formation of multiple small expansive tumour nodules, consisting of carcinoma and desmoplastic tissue. All IBC were of high histological grade: tubular formation was seen in only one case, nuclear pleomorphism was considerable and there was brisk mitotic activity.

Necrosis was not a prominent feature in IBC. Only seven cases showed small foci of necrosis in the expansive nodules of infiltrative carcinoma. Remarkably, necrosis was seen in the lymphovascular tumour emboli of eight patients. None of the IBC cases contained a fibrotic focus that is, a scar-like area replacing necrosis (Colpaert *et al*, 2001b).

### Angiogenesis-related parameters

All parameters related to angiogenesis are summarised in Table 1

**Table 1:** Angiogenesis-related parameters in inflammatory breast cancer (IBC)

**Parameter**	**IBC *n*=35**	**Non-IBC unfavourable**	**Non-IBC favourable**	*P*-value (IBC *vs* non-IBC/^*^global)
Mean Chalkley count	11.6±4.1 (11.0)	8.6±2.2 (8.6)	7.2±2.1 (7.2)	<0.0001
		*n*=46	*n*=56	^*^<0.0001
Maximal Chalkley count	13.0±4.3 (12.0)	9.6±2.6 (10)	8.0±2.4 (8.0)	<0.0001
		*n*=46	*n*=56	^*^<0.0001
Mean % immature vessels	87.3±12.6 (92.3)	88.3±10.2 (91.0)	84.0±13.7 (90.0)	>0.1
		*n*=16	*n*=18	^*^>0.1
Maximal % immature vessels	93.2±8.9 (97.0)	94.1±7.4 (97.5)	90.4±11.3 (93.0)	>0.1
		*n*=16	*n*=18	^*^>0.1
Mean ECP%	19.2±14.4 (15.3)	10.9±5.4 (8.6)	11.1±8.1 (9.0)	0.014
		*n*=16	*n*=17	^*^0.049
Maximal ECP%	23.6±17.1 (20.0)	14.5±6.5 (11.5)	14.5±10.5 (12.0)	0.04
		*n*=16	*n*=17	^*^0.11
CA IX expression	Present: 16	Present: 28	Present: 24	>0.1
	Absent: 19	Absent: 13	Absent: 29	^*^0.046
Stromal fibrin deposition	None: 15	None: 21	None: 25	0.02
	Sparse: 11	Sparse: 22	Sparse: 26	^*^0.08
	Abundant: 9	Abundant: 3	Abundant: 5	

ECP%=fraction of proliferating endothelial cells; CA IX=carbonic anhydrase IX. Continuous variables are represented as mean±s.d. (median).

.

**Figure 1:**
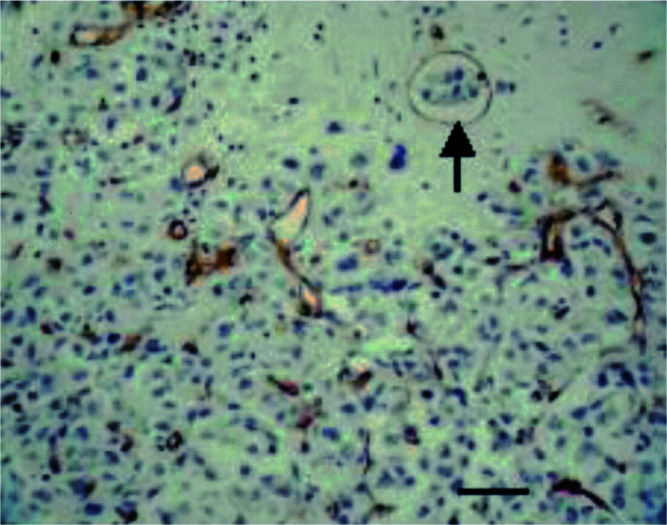
Highly vascularised area in IBC, showing tortuous and dilated vessels and vascular permeation (arrow) of tumour cells (CD 34 immunohistochemical stain). Scale bar=40 *μ*m.

Microvessel density, quantified by the Chalkley method, was significantly higher in IBC than in non-IBC (*P*<0.0001). Since the Chalkley method is an estimate of relative area, this means that the intratumoural vascular area was significantly larger. The vessels were often tortuous, branched and dilated, and vascular permeation of tumour cells was frequent (Figure 1

). The intravascular tumour emboli were never covered by endothelium.

Microvessel density (MVD), quantified by the Weidner method (Weidner *et al*, 1991), is a measurement of the number of microvessels in a microscopic field. This number was not significantly different in IBC and non-IBC: mean MVD 68.6±28.9 (median 61.2) in IBC and 72.9±21.9 (median 73.5) in non-IBC.

**Figure 2:**
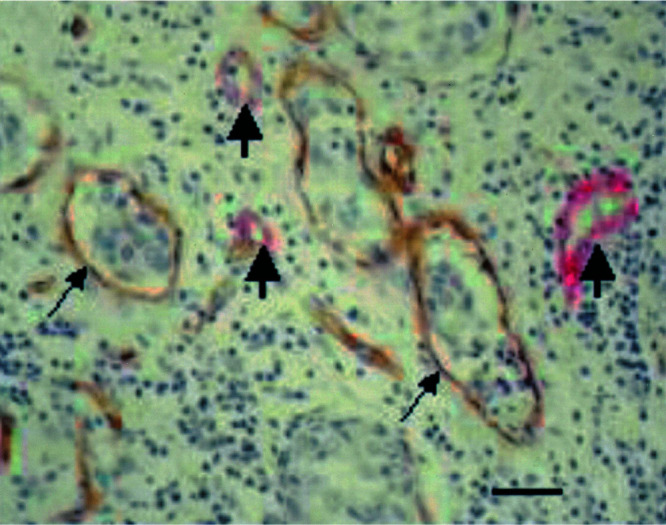
Mature vessels (thick arrows) lined by endothelial (brown) and mural cells (red) and immature vessels lined by endothelium only (thin arrows) and containing tumour emboli (CD34-*α*-smooth muscle actin immunohistochemical doublestain). Scale bar=30 *μ*m.

Although the mean percentage of immature vessels, that is, vessels consisting of endothelial cells not supported by mural cells (Figure 2

), was as high as 87.3%, this was not significantly different from non-IBC.

**Figure 3:**
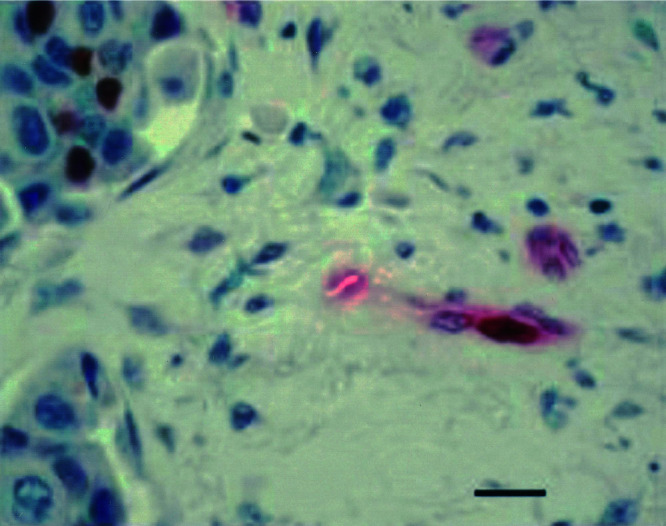
Immunohistochemical doublestain to demonstrate proliferating (brown nucleus) and nonproliferating (blue nucleus) endothelial cells (PCNA-CD34 immunohistochemical doublestain). Scale bar=18 *μ*m.

The fraction of proliferating endothelial cells (ECP%; Figure 3

) was significantly higher in IBC than in non-IBC: mean ECP% was 19% in IBC *vs* 11% in non-IBC (*P*=0.014). There was no correlation between mean ECP% and microvessel density (both Chalkley and Weidner method), mean percentage of immature vessels or stromal fibrin deposition.

**Figure 4:**
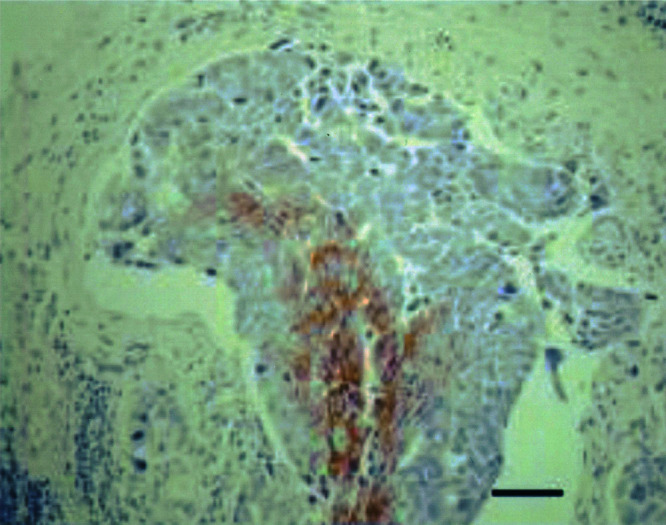
Intravascular tumour embolus, with hypoxic cells in the centre showing membranous immunoreactivity for the hypoxia marker carbonic anhydrase IX (CA IX immunohistochemical stain). Scale bar=70 *μ*m.

The endogenous hypoxia marker CA IX was expressed in only 46% of the IBC patients, while it was expressed in 68% of non-IBC patients with unfavourable prognosis (*P*=0.047) and in 45% of non-IBC patients with favourable prognosis. When present, CA IX expression in IBC was mainly observed in infiltrative carcinoma cells, although in nine patients, immunoreactivity for this hypoxia marker was also seen in intravascular tumour emboli (Figure 4

). Only three of these patients also showed necrosis in the tumour emboli.

There was a significant positive correlation between the expression of CA IX and mean ECP in IBC (*r*=0.4, *P*=0.03, 95% CI=0.04–0.7). Mean ECP% was 27.3±15.5 (26.3) when CA IX expression was present *vs* 14.8±11.2 (12.8) when CA IX was not expressed (*P*=0.04). Similarly, the maximal ECP% was 32.3±18.4 (27.5) when CA IX was present *vs* 18.9±13.9 (17.5) when CA IX was absent (*P*=0.04).

**Figure 5:**
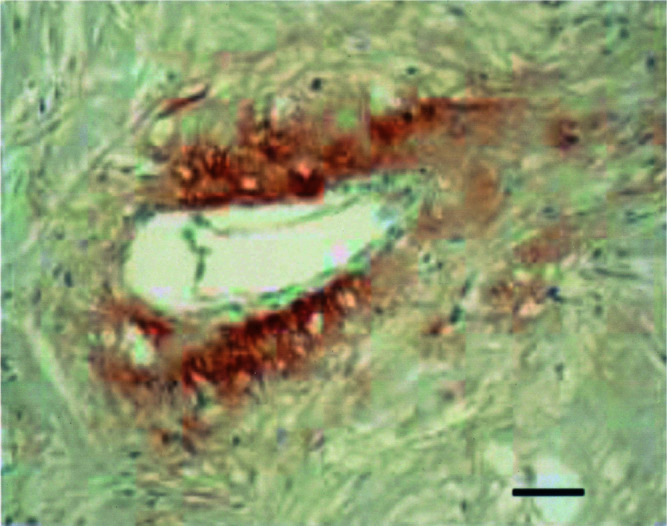
Abundant fibrin deposition in the stroma surrounding a vessel (T2G1 immunohistochemical stain). Scale bar=36 *μ*m.

Stromal fibrin depositions were found in 57% of IBC patients. These depositions were abundant in 26% of IBC patients (Figure 5

) and in only 8% of non-IBC patients (*P*=0.03).

### E-cadherin

**Figure 6:**
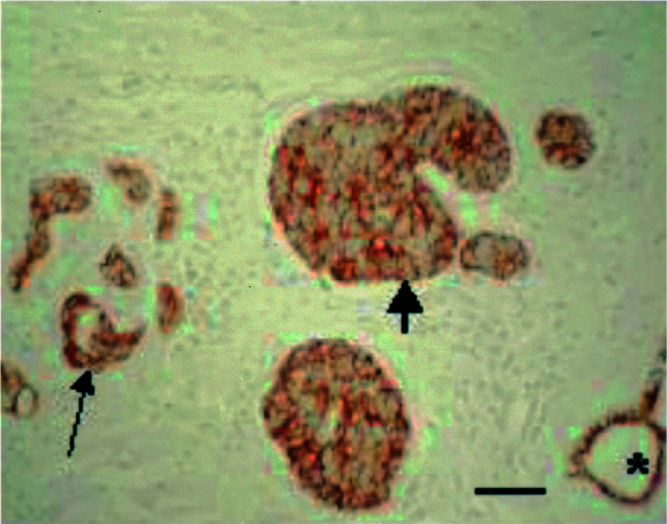
E-cadherin expression in infiltrating carcinoma cells (thin arrow), in the lymphovascular tumour emboli (thick arrow) and in a normal mammary duct (asterisk). All tumour cells show homogeneous membranous immunoreactivity at cell–cell contacts. (E-cadherin immunohistochemical stain). Scale bar=60 *μ*m.

Expression of the epithelial cell–cell adhesion molecule E-cadherin was observed in all but two of the IBC patients. The immunostaining was strikingly homogeneous, all tumour cells showing membranous immunoreactivity at cell–cell contacts (Figure 6

). In most patients, the staining intensity was equally strong in the infiltrating carcinoma cells as in the lymphovascular tumour emboli; the latter stained more intensely than the former in five cases. The staining intensity of both infiltrative carcinoma and lymphovascular tumour emboli was usually similar to that of normal epithelial structures such as the epidermis or normal mammary ducts. In five patients, expression of E-cadherin was weaker in the tumour cells than in the normal structures, but in six cases the opposite was true. No correlation was found between the intensity of E-cadherin immunostaining and the expression of the hypoxia marker CA IX.

Both carcinomas totally lacking E-cadherin expression showed a scattered growth pattern as in infiltrating lobular carcinomas, but with more pleomorphic nuclei. There was immunoreactivity at the intercellular junctions of the epidermis, serving as a positive internal control. Both patients showed the typical clinical features of inflammatory breast cancer and the diagnosis was confirmed with a skin biopsy showing dermal lymphovascular tumour emboli.

## DISCUSSION

Most of our limited knowledge about the molecular mechanisms or pathogenesis of IBC has been obtained in human IBC xenograft models. In the present study, we verified some of the findings in these models in actual cases of human IBC.

A well-studied model for IBC is the human-severe combined immunodeficient/nude mice transplantable xenograft MARY-X (Alpaugh *et al*, 1999,^2002^; Tomlinson *et al*, 2001). MARY-X consists of a confluence of tumour nodules growing in a host mesenchyme in the central part of the xenograft and tumour emboli within lymphovascular spaces at the tumour's periphery. Western blot analysis of MARY-X compared with non-IBC xenografts revealed a 10–20-fold overexpression of E-cadherin and MUC1, but markedly decreased sialyl-Lewis X/A carbohydrate ligand-binding epitopes. The latter are necessary for binding endothelial cell E-selectin, and their deficiency results in a tumour cell–endothelial cell aversion. The strong homotypic tumour cell–tumour cell adhesion mediated by the epithelial cell–cell adhesion molecule E-cadherin, together with the weak heterotypic tumour cell–endothelial cell adhesion, generates metastases in the form of compact spheroids. This phenomenon would explain the efficiency of metastatic dissemination in IBC. It has been known for several decades that haematogenous multicellular tumour cell clusters are more efficient at forming metastases than single cells (Liotta *et al*, 1976). Furthermore, tumour cell clumps are hypoxic in their centres and therefore resistant to the effects of radiation therapy and chemotherapy (Moore *et al*, 1992).

In the present study of 35 human IBC cases, lymphovascular tumour emboli were seen in all but one patient. Expression of the hypoxia marker CA IX and necrosis of the tumour emboli were seen in nine and eight patients, respectively, with only three patients showing both lymphovascular CA IX expression and necrosis. Strong E-cadherin expression was seen at the cell–cell contacts of all tumour cells in 94% of the patients. This confirms the experimental observations in MARY-X and the overexpression of E-cadherin described earlier in a limited number of human IBC cases. In our study, the immunoreactivity for E-cadherin in the lymphovascular tumour emboli was equally strong or even stronger–and never weaker–than in the infiltrative carcinoma cells. Nevertheless, the latter also showed homogeneous E-cadherin expression at cell–cell contacts in all tumour cells. It is difficult to understand how these cohesive tumour cells, strongly expressing the invasion-suppressor molecule E-cadherin (Vleminckx *et al*, 1991), can establish their highly invasive phenotype with florid lymphovascular invasion. Indeed, the current dogma is that the metastatic process requires decreased tumour cell–tumour cell adhesion, active invasion of the extracellular matrix, intravasation, extravasation and secondary growth at the metastatic site (Weidner, 2002). It has been suggested, however, that IBC bypasses some of the classic steps of metastatic progression (Alpaugh *et al*, 2002). Preliminary studies would indicate that the tumour cell embolus in IBC finds itself within the vascular lumen because it stimulates a vascular channel to form around it rather than intravasating into pre-existing lymphatics or capillaries. This mechanism shows some similarities with an invasion-independent pathway of blood-borne metastasis described earlier in a murine mammary tumour model (Sugino *et al*, 2002). In that model, the tumour nests gain access into blood vessels not through active intravasation, but because they are enveloped by vascular endothelial cells. The tumour embolus conserves its tissue organisation and endothelial covering and is mechanically arrested in a pulmonary arteriole where it proliferates intravascularly. Both in the MARY-X xenograft model of IBC and in the human IBC cases described in the present study, the intravascular tumour emboli conserve tissue organisation by maintaining a nested architecture and strong intercellular adhesion, but were never covered by endothelium. This constitutes a major difference with the invasion-independent murine mammary tumour model described above. Other recent studies have challenged the canonical steps of the metastatic process and found that haematogenous metastases originate from the proliferation of attached intravascular tumour cells rather than from extravasated ones (Al-Mehdi *et al*, 2000). Interestingly, pulmonary emboli of the MARY-X xenograft model do not extravasate either (Alpaugh *et al*, 1999).

Prerequisites for the formation of a vascular channel enveloping tumour cell nodules, as described in the invasion-independent pathway of metastasis, are high angiogenic activity and sinusoidal remodelling of tumour blood vessels (Sugino *et al*, 2002). We present strong evidence that there is indeed intense ongoing angiogenesis in human IBC. This is manifested by high Chalkley counts and high endothelial cell proliferation fractions. High Chalkley counts reflect high intratumoural vascular area. Indeed, the vessels in IBC were often tortuous, branched and dilated, thereby providing a large contact area between tumour cells and endothelial cells. This may be important for the paracrine interactions between these cells (Folkman, 1994) and may also mechanically promote the metastatic process by facilitating the entry of tumour cells into the vascular lumina. Newly formed proliferating capillaries often have fragmented basement membranes and are leaky, making them more accessible to tumour cells than mature vessels (Weidner, 2002). We showed that the mean percentage of immature vessels, defined as vessels lined by endothelial cells unsupported by mural cells, was almost 90% in the most vascularised areas of human IBC.

Despite the significantly higher Chalkley counts, microvessel density quantified by the Weidner method was not significantly higher in the IBC patients than in the non-IBC patients used for comparison. This means that the number of vessels counted in the most vascularised areas was not significantly higher. The number of vessels in a microsopic field is the result of angiogenesis, vessel remodelling and angioregression. If the latter predominates over the former, even highly angiogenic tumours can have low MVDs. Sinusoidal remodelling of vessels, as described in the invasion-independent murine mammary tumour model (Sugino *et al*, 2002), results in high intratumoural vascular area with high Chalkley counts, but low MVD according to Weidner.

The high fraction of proliferating endothelial cells (ECP) is the strongest argument for intense ongoing angiogenesis in the cases of human IBC presented in this study. There was a significant positive correlation between the expression of the hypoxia marker CA IX and ECP, implying that the angiogenesis is at least partly hypoxia driven. CA IX is one of the cell surface transmembrane carbonic anhydrases involved in maintaining an acidic extracellular pH in tumours. Its transcription is regulated by hypoxia inducible factor-1 (HIF-1), an important mediator of gene expression patterns in response to hypoxia in tumours (Wykoff *et al*, 2000). Other genes upregulated by hypoxia through activation of HIF-1 are angiogenic growth factors including VEGF (Forsythe *et al*, 1996; Jiang *et al*, 1997; Maxwell *et al*, 1997). This is a major molecular pathway for hypoxia-driven angiogenesis.

The expression of CA IX was significantly less frequent in IBC than in non-IBC, especially when compared to non-IBC patients with unfavourable prognosis. This suggests that intratumoural hypoxia is not a prominent feature of IBC and is supported by the fact that necrosis was present only focally in a minority of cases and that none of the IBC cases contained a fibrotic focus (Colpaert *et al*, 2001b). The presence of necrosis and CA IX expression in intravascular tumour emboli (Figure 4) is of interest, indicating the potentially unfavourable hypoxic microenvironment of these channels. Thus, there may be further selection for aggressive cancer cells surviving this route. The intravascular hypoxia may be because of acute intermittent vessel shut down, that is, acute hypoxia, now thought to be an important contributor to radiation and drug resistance (Harris, 2002).

The less frequent presence of CA IX in IBC *vs* non-IBC contrasts with the significantly higher ECP in IBC and points at a role for still other factors than hypoxia in stimulating angiogenesis. A possibly important oncogene product in that context is RhoC GTPase (Van Golen *et al*, 2000a,2000b), which was found to be overexpressed in 90% of archival IBC tumour samples, but not in stage-matched non-IBC tumours. High levels of VEGF, basic fibroblast growth factor, interleukin-6 and interleukin-8 were found in the conditioned media of transfectants of human mammary epithelial cells overexpressing the RhoC gene. Moreover, these transfectants were more motile with an increase in actin stress fibres and focal adhesion contact formation. These data suggest that overexpression of RhoC GTPase is a specific genetic alteration of IBC leading to the highly invasive phenotype of IBC and to the production of angiogenic factors irrespective of intratumoural hypoxia.

Indirect evidence for high VEGF production in IBC is the abundant stromal fibrin deposition observed in 26% of IBC and in only 8% of non-IBC cases. VEGF is also known as vascular permeability factor (VPF) and promotes extravasation of plasma fibrinogen, leading to fibrin deposition in the tumour matrix, which promotes the ingrowth of macrophages, fibroblasts and endothelial cells (Dvorak *et al*, 1995).

In conclusion, we showed that strong E-cadherin expression and intense ongoing angiogenesis are present in human IBC. Both features may contribute to the high metastatic efficiency of IBC. Furthermore, the intense ongoing angiogenesis demonstrates that IBC patients are a homogeneous population suffering from an angiogenesis-dependent form of breast cancer with poor prognosis. This leads to a clinically recognisable, well-defined target population for the implementation of new treatment strategies introducing combinations of cytotoxics with antiangiogenic (e.g. anti-VEGF) compounds.

Further molecular studies comparing IBC and non-IBC are needed in order to identify transcriptional and translational pathways responsible for the inflammatory carcinoma phenotype.
